# The Mediation Effect of Perceived Attitudes toward Medical Service on the Association between Public Satisfaction with the Overall Medical Service and Self-Rated Health among the General Population in China: A Cross-Sectional Study

**DOI:** 10.3390/ijerph20043369

**Published:** 2023-02-14

**Authors:** Wanwan Zheng, Yuqing Liang, Woon Seek Lee, Youngwook Ko

**Affiliations:** 1 Graduate School of Management of Technology, Pukyong National University, 365 Sinseon-ro, Nam-gu, Busan 48547, Republic of Korea; 2 Institute for Basic Science, Daejeon 34126, Republic of Korea

**Keywords:** public satisfaction with overall medical service, perceived attitudes toward medical service, mediation analysis

## Abstract

This study aimed to examine the association between public satisfaction with the overall medical service and individuals’ self-rated health among 18,852 Chinese adults aged 16–60 years by using data from the 2018 China Family Panel Studies. We further test whether such an association is mediated by perceived attitudes toward the medical service. The logistic regression model is used to explore the association between public satisfaction with the overall medical service and individuals’ self-rated health (SRH) outcomes. Mediation analysis was conducted by using the Karlson–Holm–Breen (KHB) method. We found that public satisfaction with the overall medical service was associated with good SRH. Additional results indicated that the association between public satisfaction with the overall medical service and SRH was significantly mediated by perceived attitudes toward the medical service. The degree of mediation is much larger for individuals’ satisfaction with the level of medical expertise than for trusting in doctors, attitudes toward medical service problems, and the attitude toward the level of the hospital. Targeted medical policy interventions are designed to promote individuals’ perceived attitudes toward the medical service, which might help to improve individuals’ health benefits.

## 1. Introduction

Several significant changes have been made to China’s healthcare system, greatly affecting individuals’ access to healthcare after the economic reform launched in the late 1970s [1]. Inequalities due to the increasing demand for healthcare services and limited access to basic healthcare services have exacerbated individuals’ health and wellbeing issues [2]. Accordingly, the Chinese government has launched healthcare reform actions as part of ‘the 12th Five-Year Plan’, which aims to create a basic universal healthcare system that permits individuals to use healthcare services effectively [3] and further pays special attention to increasing investment in primary care [4]. The Healthy China 2030 (HC 2030) blueprint, released in Beijing by the Chinese government, aims to improve the level of health nationwide, control major health risk factors, increase the capacity of the health service, enlarge the scale of the health industry, and perfect the health service system [5]. Nevertheless, different healthcare policies and interventions have offered substantial insights in improving healthcare services and promoting individuals’ health benefits. However, quantitative evidence of how individuals’ health benefits are affected by the subjective assessment of healthcare services is still lacking, and it needs further investigation.

Health reform implies a shift in health resources to the primary level, and primary medical and health services play an essential role in influencing medical and health work. This is deemed essential to solving the problem of expensive medical services, especially for individuals who have limited access to healthcare facilities. However, it is worth noting that it is still unknown which factors play a more important role in affecting the performance of the healthcare system [6]. Moreover, the issue of how to evaluate the performance of different levels of the medical service needs consistent evidence. Note that previous studies have used specific indicators to evaluate the performance of the health service system. For example, a study used one indicator to determine whether individuals are satisfied with health services [7]. Another study indicated that “inhabitants’ satisfaction with health service” represents “a sense of contentedness, achievement or fulfillment that results from meeting inhabitants’ needs, desires, and expectations with respect to healthcare service” [8,9]. Examining individuals’ subjective assessment of health services can help governments and policymakers to better understand individuals’ actual needs and identify the potential problems of the healthcare system.

The association between patient satisfaction with the medical service and related health outcomes has been widely studied during the past few decades. For example, one study found that patients who were satisfied with their medical service facilities were more likely to report better survival outcomes compared to those who did not. They further found that SRH played an independent role in influencing the association between patient satisfaction with medical service quality and survival outcomes [10]. Other studies indicated consistent findings between patients’ satisfaction with medical services and survival outcomes, such as in breast [11], colorectal [12], and non-small-cell lung cancer patients [13]. However, unlike studies focusing on patients’ satisfaction, public satisfaction has been considered one of the most reliable indicators of the general subjective evaluation of the healthcare system [14]. On one hand, it provides a comprehensive investigation of satisfaction with the overall medical service for both patients and non-patients of the medical service. On the other, it directly demonstrates how the general population uses medical services and whether they trust in the healthcare system [15]. Understanding the relationship between public satisfaction with medical services and individuals’ SRH can have essential implications in forming appropriate healthcare policy evaluations to promote individuals’ health benefits [16,17].

Individuals’ subjective assessments of medical services play an essential role in influencing people’s health outcomes. Various studies have shown that patients’ satisfaction with medical care is associated with various health outcomes. They highlight the importance of patients’ overall satisfaction in affecting the knowledge of patients about the doctor, their technical care, and practice nursing that potentially contribute to patients’ health benefits [18]. One study conducted in the Vilnius area in Lithuania found that respondents with a higher level of satisfaction with primary healthcare services were associated with less depression [19]. Furthermore, a mixed association has been found between trust in doctors and individuals’ health benefits. Studies have indicated that complex doctor–patient relationships have further stimulated people’s interest in public satisfaction [3,20]. The patient’s distrust in the doctor aggravated the tension in the doctor–patient relationship and reduced the patient’s trust in the doctor, which resulted in dissatisfaction with the quality of the medical service [21]. One study, for example, found a significant association between trust in doctors and mental health symptoms [22]. Another study indicates a curvilinear association between trust in doctors and anxiety when respondents consult a doctor [23]. One underlying mechanism for such an association might be that patients perceived their physician to be knowledgeable and trustworthy, which contributed to their satisfaction [24].

Numerous studies have indicated consistent findings between individuals’ sociodemographic characteristics and self-rated health outcomes. For instance, studies have found consistent results showing that older adults were more likely to report poor SRH compared to their younger counterparts [25]. Respondents who were female, married, and unemployed were more likely to report poor SRH [26,27,28]; on the other hand, respondents with a higher educational level and higher income level were more likely to report good SRH [29,30]. In addition, rural residents (without hukou) are more likely to report poor SRH outcomes than those with urban hukou [31]. Regarding individuals’ health risk characteristics, individuals with sufficient physical activity levels tend to report good SRH compared to those who do not satisfy these criteria [32]. However, individuals who reported that they had chronic diseases, or were current smokers or drinkers, tended to have higher odds of reporting poor SRH [33,34]. Obese respondents tended to have higher odds of reporting health outcomes than those with a normal weight [35]. Additionally, spatial heterogeneity between public satisfaction with the overall medical service and SRH, which is affected by an individual’s sociodemographic and health risk characteristics, should be noted and warrants further consistent study [36].

Despite the growing interest in examining the association between individuals’ satisfaction with medical services and individuals’ health benefits, most of these studies were conducted based on a small survey sample. Moreover, no previous studies have systematically explored the association between public satisfaction with the medical service and individuals’ self-rated health based on a nationwide database. Therefore, this study aims to explore the association between public satisfaction with the medical service and individuals’ SRH among the general population. This study further explores the potential mediation role of perceived attitudes toward medical services on such an association. This study contributes to the previous literature by providing novel insights by directly examining how individuals’ perceptions of the medical service would play an important role in affecting individuals’ health benefits. Accordingly, three research questions were generated:

(1) Are individuals who are more satisfied with the overall medical service more likely to report better self-rated health outcomes compared to those who do not?
(2) Do individuals’ perceived attitudes toward the medical service play a significant mediating role in influencing the association between satisfaction with the overall medical service and self-rated health outcomes?
(3) Is the association between satisfaction with the overall medical service and self-rated health outcomes moderated by individuals’ sociodemographic characteristics?

The remainder of this paper is organized as follows. Section 2 outlines the data and methods utilized in this study. Section 3 presents the results. Section 4 discusses the main findings and reveals the practical implications of the study. Section 5 concludes the study.

## 2. Materials and Methods

### 2.1. Sample Description

Longitudinal data from the China Family Panel Studies (CFPS) were obtained to explore the influences of satisfaction with healthcare services on individuals’ SRH. Of note, CFPS covers 25 provinces or their administrative equivalents in China and includes 95% of the Chinese population, which can be considered as a nationally representative sample [37]. CFPS surveys have been conducted by Peking University every two years since 2010, and the most recent wave (2018) was released in 2019. The Peking University Biomedical Ethics Review Committee provided ethical approval for the survey (approval number: IRB00001052-14010) [38]. All the respondents read a statement that explained the purpose of the study and gave consent to continue. CFPS surveys are composed of five parts: the community questionnaire, the family roster questionnaire, the family questionnaire, the child questionnaire, and the adult questionnaire.

In this study, the latest wave of CFPS data (2018) was used to explore the aims and objectives of the study, since it provided a sufficient set of questions to assess an individual’s related health benefits and sociodemographic characteristics. Overall, 32,669 samples were captured after matching the database of the adult questionnaire and family questionnaire and further deleting the duplicate samples. Listwise deletion for missing data on the variables was conducted in the analysis. Specifically, we restricted our sample to adult respondents aged between 16 and 60 years due to the needs of the study, followed by excluding the samples with the responses of “I don’t know”, “not applicable”, “I refuse to answer”, and missing values. Finally, a sample size of 18,852 was obtained to estimate the influences of public satisfaction with the overall medical service on individuals’ SRH.

### 2.2. Variable Definition

#### 2.2.1. Measures

Previous studies have widely used SRH to explore individuals’ health status [39,40]. SRH was generally measured by the question “how would you rate your health status?”. The traditional format of SHR, proposed by the WHO (1996), indicated the following scale: “very good”, “good”, “fair”, “bad”, “very bad”. In contrast, in this study, the response to the question used a five-point Likert-based scale, reported as (1) “excellent”, (2) “very good”, (3) “good”, (4) “fair”, (5) “poor”. In order to reflect subjective measures of health status, we further converted it into a dummy variable, defining SHR with values of (1) to (4) as good SRH, while the value of (5) indicated poor SRH. Dichotomizing SRH has been commonly used as an effective approach in the previous literature [41,42].

#### 2.2.2. Key Independent Variables

One of the key independent variables considered in this paper is individuals’ satisfaction with the overall medical service. In this study, individuals’ satisfaction with the medical service was measured by the following question: “Are you satisfied with the overall medical service?”. Here, “medical service” refers to the conditions of doctors, medicine, hospitalizations, etc. It also includes the travel distance and transportation convenience. Response categories ranged from (1) very satisfied to (5) very unsatisfied. We further reversed and coded the item so that it ranged from (1) very unsatisfied to (5) very satisfied.

#### 2.2.3. Mediators

In this study, our focal mediator variable is individuals’ perceived attitudes toward the medical service, which includes four aspects. All indicators related to perceived attitudes toward the medical service were derived from the China Family Panel Studies 2018 Full Questionnaires, which can be directly downloaded from https://opendata.pku.edu.cn/dataverse/CFPS?language=en (accessed on 2 January 2022). Descriptions of each type of related question can be derived from the China Family Panel Studies 2018 Full Questionnaires (https://www.isss.pku.edu.cn/cfps/docs/20210812133818010131.pdf?CSRFT=JZIO-YGCJ-X8EZ-2YN9-H9TP-6P3K-TY22-VV5A (accessed on 2 January 2022)).

Specifically, the attitude toward the medical service problems was measured with the question “How would you rate the severity of the medical service problem in China?”. Response categories ranged from (0) no problem to (10) extremely serious problems. We reversed and coded the item according to its ordinal attributes. Trust in doctors was measured with the question “How much do you trust doctors?” Response values ranged from 0 (“distrustful”) to 10 (“very trusting”). Respondents’ attitudes in terms of medical expertise was measured with the question “What do you think of the medical expertise level?” Response values ranged from 1 (“very bad”) to 5 (“very good”). The attitude toward the level of the hospital was measured with the question “Where would you usually go to see a doctor?” Note that the classification of Chinese hospitals is a 5-tier system according to the CFPS2018 Adult Questionnaire. China has classified its hospitals since at least 2008 according to a system that recognizes medical care, medical education, and medical research capabilities. Accordingly, response categories were rated using a five-point Likert-based scale, reported as (1) “general hospital”, (2) “specialty hospital”, (3) “community healthcare center/township hospital”, (4) “community healthcare post/village clinic”, (5) “clinic”. Specifically, “general hospital” refers to the so-called “big hospital”, where various types of diseases can be diagnosed and treated. “Specialty hospital” refers to the hospitals that pay special attention to specific types of diseases, such as a hospital for obstetrics and gynecology. “Community healthcare center/township hospital” refers to a medical facility established at the urban community or township level to treat common diseases. “Community healthcare post/village clinic” refers to a medical facility established at the urban community or village level to treat common diseases. “Clinic” refers to a private clinic, as well as a poorly equipped community/village clinic. We reversed and coded the item according to its ordinal attributes.

#### 2.2.4. Covariates

Previous studies have documented that individuals’ physical health status, physical activity, and daily behaviors are significantly associated with SRH [43,44,45]. Accordingly, we explored five related health risk factors that would play an important role in affecting SRH, namely body mass index (BMI), chronic illness, physical activity, and being a current smoker or drinker. Respondents’ BMI was measured with a question asking about respondents’ current height and weight. The response was a continuous variable. Respondents’ chronic status was measured with the question “During the past six months, do you have any doctor-diagnosed chronic disease?” The answer was coded as (1) yes or (0) no. Respondents’ frequency of physical activity was measured with the question “how often did you participate in physical exercise in the past week?” The response was a continuous variable. Smoking status was measured with the following question: “Did you smoke cigarettes in the past month?” The answer was coded as (1) yes or (0) no. We measured drinking status with the following question: “Did you drink alcohol at least 3 times per week in the past month?” This item was coded as (1) for drinkers or (0) otherwise. Finally, we adjusted for individuals’ sociodemographic characteristics, such as age, gender, marital status, educational attainment level, household income level, family size, employment status, hukou status, and residence in an urban area, or having medical insurance. Note that “hukou status” refers to a salient social identity in China where respondents who live in urban areas are categorized as urban residents while respondents living in rural areas are categorized as rural residents. The hukou system plays a substantial role in controlling the movement of the population and individuals’ social and economic outcomes, which exacerbates the inequality of access to urban resources between urban and rural populations [46]. Economic resources, education, employment, and social welfare benefits are biased toward urban residents [46].

### 2.3. Analytic Strategy

The associations between satisfaction with the overall medical service and SRH (Model 1) were first assessed as a crude model by using the logistic regression model in order to answer our first research question. We further adjusted for mediators and related health risk factors in Model 2 and Model 3, respectively. Finally, we adjusted for sociodemographic characteristics in Model 4. For the analytical sample, all models were fitted without missing data to ensure differences between them that were not due to different participants being included.

To formally answer our second research question, we introduced the KHB method to examine the total, direct, and indirect associations between public satisfaction with the overall medical service through individuals’ satisfaction, where individuals’ satisfaction included the level of medical expertise, attitudes toward medical service problems, trust in doctors, and the attitude toward the level of the hospital [47,48,49]. This method has been widely conducted in terms of accessing the influence of individuals’ sociodemographic characteristics and their health and wellbeing [50,51,52]. Note that the estimated effects of the reducing model refer to the total effect, since it did not include the mediator, while the estimated effect of the full model refers to the direct effect. The difference between these models was calculated using the KHB method as the indirect association between public satisfaction with the medical service and SRH through mediators.

**Figure 1:**
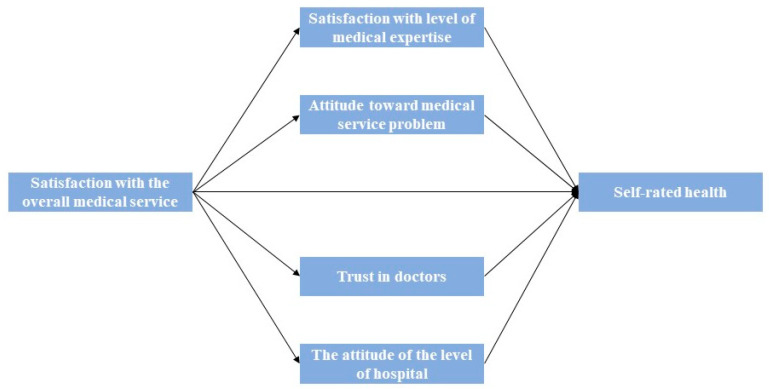
Mediation of the association between public satisfaction with the overall medical service and SRH.

The technical details of the KHB method can be found in previous studies [47]. One essential advantage of using the KHB method is that the KHB method can handle multiple mediators simultaneously [47]. The KHB method can ascertain which of the mediators play the most essential role in contributing to the confounding [48]. Figure 1 illustrates the mediation process of the association between public satisfaction with the overall medical service and SRH. Following the KHB method, derived from the study, the total, direct, and indirect associations between satisfaction with the overall medical service and SRH through perceived attitudes toward the medical service were assessed.

Logistic regression and KHB methods were performed by using STATA version 16. We applied odds ratios (ORs) with 95% confidence intervals (CI) to present the estimation results. Note that results from the KHB method were reported with regression coefficients and standard errors. Finally, we applied the variation inflation factor (VIF) to explore the potential collinearity between individuals’ satisfaction with the overall medical service, mediators, and SRH. The results indicated that there were no collinearity issues in the estimation model because none of the VIFs were greater than 2.1.

## 3. Results

Table 1 shows the baseline factors (N = 18,852) of the current study. We found that 85% of the respondents reported a good SRH. Note that 12.2% of them reported having a chronic disease. Moreover, 51% were male respondents, while 49% were female. Additionally, around four in five of all respondents were married, and 1.4% of the respondents were retirees. Furthermore, 24.5% and 51.4% had hukou status and were registered as urban residents, respectively. The average age of the respondents was 41 years. Nearly 90% were employed and 77% had an educational attainment level of junior high school and above. The average total income of the respondents was CNY 22,359 per year, with only 2.3% having medical insurance. In terms of health risk characteristics, the average frequency of physical exercise for respondents was two times per week, which is in line with reporting a normal weight for the general population in Asian countries, with a BMI value of 23.3 [53]. In addition, we found that nearly two fifths and more than 16% of respondents were current smokers and had consumed alcohol at least three times per week in the previous month. Regarding the subjective assessment of medical services, the survey found that 66% of respondents trusted their doctors, and half of the respondents were pleased with how hospitals provided medical services and the medical expertise that they provided. Moreover, 50% of the respondents had a neutral attitude toward medical service problems and over half of the respondents preferred to visit the community healthcare center/township hospital, a specialty hospital, or a general hospital to see a doctor, instead of visiting a community healthcare post or clinic.

### 3.1. Association between Factors and Respondents’ SRH

Table 2 presents the results from the logistic regression model estimating the association between independent variables and individuals’ good SRH. We found that increased satisfaction with the overall medical facilities was associated with higher odds of reporting good SRH (OR = 1.120, 95% CI 1.063–1.181, *p* < 0.01). Such influence remains robust after adjustment for covariates (OR = 1.096, 95% CI 1.021–1.176, *p* < 0.05).

Regarding the perceived attitudes toward the medical service, respondents with a decreased level of attitude toward medical service problems were associated with good SRH after full adjustment (Model 4: OR = 1.098, 95% CI 1.026–1.174, *p* < 0.01). Respondents who expressed satisfaction with the level of medical expertise also had higher odds of reporting good SRH (2: OR = 1.107, 95% CI 1.104–1.178, *p* < 0.01). Such an association was stronger after adjustment for related health risk factors in Model 3 (OR = 1.052, 95% CI 1.039–1.185) but not in Model 4 (OR = 1.098, 95% CI 1.026–1.174, *p* < 0.01). The level of trust in doctors was associated with higher odds of reporting good SRH (Model 4: OR = 1.034, 95% CI 1.013–1.055, *p* < 0.01). The level of the hospital was associated with lower odds of reporting good SRH across all models (Model 4: OR = 0.881, 95% CI 0.853–0.910, *p* < 0.01).

In our analysis of related health risk factors, exercise frequency was associated with higher odds of reporting good SRH across most analyses (Model 4: OR = 1.039, 95% CI 1.022–1.056, *p* < 0.01). A longer sleep duration was associated with lower odds of reporting good SRH (Model 4: OR = 0.850, 95% CI 0.738–0.978, *p* < 0.05). We found evidence that alcohol intake was associated with higher odds of reporting good SRH (Model 4: OR = 1.403, 95% CI 1.199–1.642, *p* < 0.01), while no significant relationship could be observed in regard to smoking behavior and good SRH associations. Respondents with chronic diseases had lower odds of reporting good SRH and such an association became more pronounced after adjustment for respondents’ covariates (Model 4: OR = 0.191, 95% CI 0.171–0.213, *p* < 0.01). There was no evidence of an association between BMI and respondents’ SRH.

Regarding the sociodemographic characteristics, men had lower odds of reporting poor SRH (Model 4: OR = 1.316, 95% CI 1.133–1.528, *p* < 0.01). Increased age was associated with lower odds of reporting poor SRH (Model 4: OR = 0.951, 95% CI 0.945–0.956, *p* < 0.01). Individuals with a high income, local hukou, medical insurance, and a full-time employment status had higher odds of reporting good SRH (See Table 2). Individuals living in urban areas had higher odds of reporting good SHR (Model 4: OR = 1.280, 95% CI 1.146–1.430, *p* < 0.01). Family sizes and individuals’ educational attainment level were associated with higher odds of reporting good SRH (Model 4: OR = 1.062, 95% CI 1.035–1.090, *p* < 0.01; Model 4: OR = 1.181, 95% CI 1.058–1.319, *p* < 0.01). We found no evidence of an association between individuals’ marital status and SRH.

### 3.2. Association between Factors and Individuals’ SRH

To comprehensively test the proposed mediating mechanisms, the KHB method was applied to examine whether the association between public satisfaction with the overall medical service and SRH was medicated by mediators.

Table 3 indicated that public satisfaction with the overall medical service increased the log odds of good SRH by 0.17. The effects of satisfaction with the overall medical service were reduced to 0.08, leaving an indirect effect of 0.08 after controlling for all mediators. Table 4 suggests that the total effect was 1.94 times larger than the direct effect and 48% of the total effect was due to the mediators. Additional results indicated that individuals’ satisfaction with the level of medical expertise had the largest mediation effect size, followed by trust in doctors, the attitude toward medical service problems, and the attitude toward the level of the hospital.

### 3.3. Stratified Analysis

We further conducted the stratified analysis across individuals’ sociodemographic characteristics for the robustness check to examine our last research question (see Table 5). We found that public satisfaction with the overall medical service was associated with higher odds of reporting good SRH in terms of males and individuals who had local hukou. Additional results evidenced that public satisfaction with the overall medical service was associated with higher odds of reporting good SRH only in individuals who had a higher educational attainment level (OR = 1.103, 95% CI 1.015–1.199, *p* < 0.05), higher income level (OR = 1.172, 95% CI 1.030–1.335, *p* < 0.05), an age over 40 (OR = 1.092, 95% CI 1.006–1.185, *p* < 0.05), and residence in a rural area (OR = 1.159, 95% CI 0.999–1.371, *p* < 0.10). These results largely echoed the findings in our baseline model.

## 4. Discussion

In implementing the Healthy China 2030 (HC 2030) blueprint, in recent decades, the Chinese government has gradually launched a series of national healthcare strategies. Improving the health service capacity and the health service system are the main foci among the strategies. However, limited attention has been paid to exploring how individuals’ self-subjective assessments of medical services would contribute to health benefits among China’s general population. This is especially true for populations with limited access to public health facilities. The contributions of this study are threefold. Firstly, we explored the association between public satisfaction with the overall medical service, perceived attitudes toward the medical service, related health risk factors, sociodemographic characteristics, and SRH. Secondly, we further examined whether individuals’ perceived attitudes toward the medical service play a significant mediating role in influencing such an association. Finally, we tested the model sensitivity by applying the stratified analysis across individuals’ sociodemographic characteristics. Findings from this study provide a better understanding of how public satisfaction with the overall medical service plays an essential role in shaping people’s health benefits, where perceived medical attitudes in public satisfaction play a mediating role in medical service–SRH associations.

The results of our baseline model revealed that increased satisfaction with the overall medical facilities was associated with higher odds of reporting good SRH. This finding sheds light on the importance of considering individuals’ subjective assessments of medical services in affecting individuals’ health benefits. This result is consistent with the finding that satisfaction with healthcare was significantly associated with SRH [70,71]. This is promising since it indicates that medical facilities achieving high individual satisfaction may contribute to obtaining good subjective overall health benefits, which provides new insights for governments to consider adjusting the medical system. Regarding individuals’ perceived attitudes toward the medical service, positive associations among individuals’ attitudes toward medical service problems, satisfaction with the level of medical expertise, level of trust in doctors, and SRH have been observed. The negative association between the level of the hospital and good SRH suggested that individuals were more likely to report poor SRH if they choose to access a larger hospital instead of smaller ones. One possible explanation might be that individuals’ satisfaction scores might be lower in a higher level of hospital. It might be influenced by individuals’ perceptions of hospital cleanliness, promptness of assistance, and communication between doctors and patients [72].

Regarding the health risk factors and individuals’ sociodemographic characteristics, we found a positive association between the frequency of physical exercise and SRH, which is consistent with the findings of other studies [73]. Individuals who had a longer sleep duration were less likely to report good SRH [74]. Interestingly, we found a positive association between alcohol intake and SRH, where this finding is consistent with several findings that more frequent alcohol intake was associated with good health [75], though not all [52,76,77]. Individuals with chronic diseases tended to report poor SRH [53]. Furthermore, men demonstrated a lower probability of reporting poor SRH than women. This finding is in line with several studies [78,79] but contradicted other studies indicating that women rated their health status more favorably than men [80]. Increased age was negatively associated with SRH, which was broadly aligned with previous studies [81]. Individuals with high income levels and medical insurance were more likely to report good SRH, which was widely in line with previous studies [82,83]. Notably, we found that individuals with local hukou and living in urban areas were more likely to report good SRH [36,84]. One possible explanation might be that the urban–rural divergence exacerbated the inequality of individuals’ access to medical facilities, especially for individuals living in rural China, where healthcare facilities are lacking, which results in spatial health disparities [85].

Regarding the findings from the mediation analysis, we found that the association between public satisfaction with the overall medical facilities and SRH was significantly mediated by perceived attitudes toward the medical service. Specifically, we found that individuals’ satisfaction with the level of medical expertise and trust in doctors play the most mediating roles in the association between individuals’ satisfaction with the overall medical facilities and SRH. This finding sheds light on the importance of healthcare service providers’ attitudes toward individuals, which are of primary importance for policymakers to promote individuals’ health benefits. The results of the stratified analysis were largely in line with the findings from our baseline model. We found that individuals’ satisfaction with the overall medical service was positively associated with SRH and such effects were more pronounced for older adults aged over 60. One possible explanation might be that vulnerable individuals such as older adults are more likely to be sensitive to the overall quality of the medical service, as the quality of the healthcare service plays a substantial role in affecting their health benefits [86]. Additional results demonstrated significant associations between individuals’ satisfaction with the overall medical service and SRH with regard to individuals with a higher socioeconomic status (SES). This finding was largely consistent with studies indicating that individuals with higher SES were more likely to report good SRH compared to their peers [87,88].

Regarding the practical implications of this study, our findings not only provide substantial pathways for stakeholders to take efforts in balancing medical resource allocation across the country, but also highlight the importance of considering individuals’ perceived attitudes toward the medical service. Primary attention regarding the medical service revolution should be paid to vulnerable groups such as older adults and children, who have limited access to medical services. Special community-based eldercare and childcare medical services are encouraged to be allocated in deprived areas to promote their health benefits. Experienced clinicians are encouraged to work in rural hospitals for a certain period in order to improve individuals’ satisfaction with the level of medical expertise [89]. Policymakers should allocate medical services appropriately across cities in accordance with individuals’ SES, to avoid the unnecessary waste of medical resources.

## 5. Conclusions

This study aimed to explore the association between public satisfaction with the overall medical service and individuals’ SRH among the general population, and additionally explored the potential mediating role of perceived attitudes toward medical services on such an association. The logistic regression model was used to examine the association between public satisfaction with the overall medical service and SRH. The KHB method was used to explore the potential mediating role of perceived attitudes toward medical services on public satisfaction with overall medical service–SRH associations. Results indicated that increased satisfaction with the overall medical facilities was associated with higher odds of reporting good SRH after adjustment for covariates. Respondents with a decreased level of attitude toward medical service problems were associated with good SRH after adjustment for covariates. Respondents who expressed satisfaction with their level of medical expertise had higher odds of reporting good SRH, though such an association was less profound after adjustment for related health risk factors and sociodemographic characteristics. The level of trust in doctors was associated with higher odds of reporting good SRH. The level of the hospital was associated with lower odds of reporting good SRH after adjustment for covariates. Additional results indicated that individuals’ satisfaction with the level of medical expertise had the largest mediation effect size, followed by trust in doctors, the attitude toward medical service problems, and the attitude toward the level of the hospital. Policymakers might need to pay special attention to ascertaining the potential pathway to improve individuals’ perceived attitudes toward medical services in order to promote individuals’ health benefits.

Although this study provides potential insights indicating that individuals’ subjective assessments of overall medical services can inform healthcare interventions and policies, limitations should not be neglected in promoting health benefits among the general population. Firstly, the causal relationship between different variables and SRH cannot be fully detected due to the cross-sectional nature of the data. The potential reverse causality, such as poor SRH, which leads to less favorable sociodemographic characteristics, living in more deprived rural areas, and an unhealthy lifestyle, could be comprehensively examined if longitudinal studies were available. Also, recall bias and selection bias might not be avoided as the nature of cross-sectional study design, which might lead to underestimation of public satisfaction with the overall medical service–SRH association. Secondly, the estimation results might be biased, as many independent variables were self-reported. Future studies should pay special attention to examining specific health indicators once the data are available. Nonlinear associations between perceived attitudes toward medical services and health benefits are encouraged to explore findings among different population groups by conducting machine learning and deep learning approaches [56,90].

## Figures and Tables

**Table 1:** Summary of the baseline factors assessed in this study.

Variable	Definition	Reference	Mean	Standard Deviation
Self-rated health	1 = good SRH, 0 = poor SRH	Leng [54], Ma, Wang and Liu [38]	0.881	0.323
Public satisfaction with the overall medical service	Ordinal scale, (1) very unsatisfied to (5) very satisfied	Franks, Gold and Fiscella [40], Chen, et al. [55]	3.586	0.813
Attitude toward medical service problems	Ordinal variable, (0) extremely serious problem to (10) no problem	Liang et al. [56], Li and Khan [57]	3.107	2.607
Trust in doctors	Ordinal variable, (0) distrustful to (10) very trusting	Meng et al. [58], Li and Khan [57]	6.631	2.383
Satisfaction with level of medical expertise	The level of medical expertise, ordinal variable 1 (very bad) to 5 (very good)	Li and Khan [57]	3.453	0.875
The attitude toward the level of hospital	The level of the hospital, ordinal variable 1 (clinic level) to 5 (general hospital level)	Zhou et al. [59]	3.092	1.615
Age	Continuous variable (years)	Ma, Wang and Liu [38]	41.079	11.256
Gender	1 = male, 0 = female	Ma, Wang and Liu [38]	0.515	0.500
Urban	1 = urban resident, 0 = rural resident	Ma, Wang and Liu [38], Meng and Chen [60]	0.514	0.500
Education	1 = junior high school and above, 0 = otherwise	Ma, Wang and Liu [38], Hu and Hibel [61]	0.772	0.420
Income	Total income, continuous variable (yuan)	Ma, Wang and Liu [38]	22,359.32	34,931.82
Marital status	1 = married, 0 = otherwise	Xie and Hu [37]	0.778	0.415
Hukou	1 = urban hukou, 0 = non-urban hukou	Xu and Xie [62]	0.245	0.430
Employment	1 = employed, 0 = otherwise	Xu and Xie [62]	0.897	0.304
Smoking	1 = current smoker, 0 = non-current smoker	Chen and Meng [63]	0.367	0.482
Drinking	1 = current drinker, 0 = non-current drinker	Chen and Meng [63]	0.158	0.365
Exercise	Frequency of physical exercise, continuous variable (times)	Dong [64], Li et al. [65]	2.211	2.951
Retirement	1 = retiree, 0 = otherwise	Yang et al. [66]	0.014	0.118
Insurance	1 = respondent had medical insurance, 0 = otherwise	Ma, Wang, and Liu [38]	0.023	0.149
Chronic illness	1 = had a chronic disease, 0 = no chronic disease	Ma, Wang and Liu [38], Zou et al. [67]	0.122	0.327
Family size	Continuous variable	Lyu and Sun [68]	4.246	2.049
BMI	Continuous variable	Chen and Meng [63], Zhou et al. [69]	23.295	3.426

**Table 2:** Logistics regression model predicting good SRH with odds ratio (OR) and 95% confidence intervals (CI).

	Model 1	Model 2	Model 3	Model 4
	OR (95% CI)	OR (95% CI)	OR (95% CI)	OR (95% CI)
Satisfaction with the overall medical service	1.120 ***	1.038	1.036	1.096 **
	[1.063,1.181]	[0.972,1.109]	[0.967,1.110]	[1.021,1.176]
Satisfaction with level of medical expertise		1.107 ***	1.110 ***	1.098 ***
		[1.041,1.178]	[1.039,1.185]	[1.026,1.174]
Attitude toward medical service problems		0.989	0.989	1.026 ***
		[0.972,1.006]	[0.971,1.007]	[1.007,1.045]
Trust in doctors		1.027 ***	1.026 **	1.034 ***
		[1.008,1.047]	[1.006,1.047]	[1.013,1.055]
The attitude toward the level of hospital		0.893 ***	0.938 ***	0.881 ***
		[0.869,0.918]	[0.910,0.966]	[0.853,0.910]
Smoking			1.093 *	0.971
			[0.986,1.211]	[0.835,1.130]
Drinking			1.481 ***	1.403 ***
			[1.274,1.722]	[1.199,1.642]
Exercise			1.020 **	1.039 ***
			[1.004,1.036]	[1.022,1.056]
Sleep duration			0.823 ***	0.850 **
			[0.720,0.941]	[0.738,0.978]
Chronic illness			0.143 ***	0.191 ***
			[0.129,0.159]	[0.171,0.213]
BMI			0.988 *	1.003
			[0.974,1.001]	[0.988,1.017]
Insurance				1.838 ***
				[1.364,2.477]
Age				0.951 ***
				[0.945,0.956]
Gender				1.316 ***
				[1.133,1.528]
Urban residence				1.280 ***
				[1.146,1.430]
Education				1.181 ***
				[1.058,1.319]
Income				1.000 ***
				[1.000,1.000]
Marital status				1.039
				[0.895,1.205]
Hukou				1.392 ***
				[1.204,1.610]
Employment				1.721 ***
				[1.489,1.990]
Family size				1.062 ***
				[1.035,1.090]
N	18,852	18,852	18,852	18,852
AIC	13,716.17	13,643.43	12,317.59	11,495.93
BIC	13,731.86	13,690.5	12,411.72	11,668.51
Pseudo R2	0.001	0.007	0.105	0.166

Note: OR (odds ratio), 95% CI (confidence interval) in brackets. * *p* < 0.10, ** *p* < 0.05, *** *p* < 0.01. AIC refers to Akaike’s information criterion. BIC refers to Bayesian information criterion.

**Table 3:** The overall KHB mediation analysis results.

SRH	Coef.	Std. Err.	z	*p* > |z|	[95% Conf. Interval]
Public satisfaction with the overall medical service					
Total effects	0.173	0.029	6.04	0.000	0.116–0.229
Direct effects	0.089	0.036	2.49	0.013	0.019–0.161
Indirect effects	0.083	0.022	3.83	0.000	0.040–0.126

**Table 4:** KHB mediation analysis to determine contributions of mediators.

	Coef.	Std. Err.	P_Diff	P_Reduced
Public satisfaction with the overall medical service				
Trust in doctors	0.021	0.007	26.06	12.52
Satisfaction with level of medical expertise	0.057	0.021	69.30	33.28
Attitude toward medical service problems	0.008	0.003	9.50	4.56
Attitude toward the level of hospital	−0.004	0.002	−4.87	−2.34

**Table 5:** Stratified analysis across individuals’ sociodemographic characteristics.

	Male	Female	Living in Urban Areas	Living in Rural Areas	With *Hukou*	Without *Hukou*
	OR (95% CI)	OR (95% CI)	OR (95% CI)	OR (95% CI)	OR (95% CI)	OR (95% CI)
SRH						
Public satisfaction with the overall medical service	1.110 **	1.088 *	1.022	1.159 ***	1.170 *	1.078 *
	[1.003,1.227]	[0.985,1.202]	[0.918,1.137]	[1.055,1.274]	[0.999,1.371]	[0.996,1.167]
N	9707.000	9145.000	9689.000	9163.000	4623.000	1.4 × 10^4^
AIC	5297.579	6178.659	5153.953	6332.274	2328.715	9169.574
BIC	5448.372	6328.199	5304.706	6481.855	2463.929	9328.398
Pseudo R2	0.143	0.181	0.156	0.168	0.148	0.168
Full controls	Y	Y	Y	Y	Y	Y
Continue	Age ≤ 40	Age > 40	With a higher income level	With a lower income level	With higher education attainment level	With lower education attainment level
	OR (95% CI)	OR (95% CI)	OR (95% CI)	OR (95% CI)	OR (95% CI)	OR (95% CI)
SRH						
Public satisfaction with the overall medical service	1.054	1.092 **	1.172 **	1.064	1.103 **	1.076
	[0.916,1.213]	[1.006,1.185]	[1.030,1.335]	[0.978,1.158]	[1.015,1.199]	[0.940,1.231]
N	8753.000	10,009	7924.000	10,928	14,547	4305.000
AIC	3367.029	8127.733	3561.096	7907.885	8464.775	3052.857
BIC	3508.572	8279.357	3707.627	8061.165	8624.063	3186.575
Pseudo R2	0.100	0.135	0.102	0.168	0.160	0.175
Full controls	Y	Y	Y	Y	Y	Y

Note: OR (odds ratio), 95% CI (confidence interval) in brackets. * *p* < 0.10, ** *p* < 0.05, *** *p* < 0.01. AIC refers to Akaike’s information criterion. BIC refers to Bayesian information criterion.

## Data Availability

Original data can be accessed from https://opendata.pku.edu.cn/dataverse/CFPS?q=&types=files&sort=dateSort&order=asc (accessed on 2 January 2022) after obtaining permission from Peking University Open Research Data.
